# Prostaglandin D_2_ and leukotriene E_4_ synergize to stimulate diverse T_H_2 functions and T_H_2 cell/neutrophil crosstalk

**DOI:** 10.1016/j.jaci.2014.09.006

**Published:** 2015-05

**Authors:** Luzheng Xue, Joannah Fergusson, Maryam Salimi, Isabel Panse, James E. Ussher, Ahmed N. Hegazy, Shân L. Vinall, David G. Jackson, Michael G. Hunter, Roy Pettipher, Graham Ogg, Paul Klenerman

**Affiliations:** a Oxford NIHR Biomedical Research Centre, Translational Immunology Laboratory, Nuffield Department of Medicine, John Radcliffe Hospital, University of Oxford, Oxford, United Kingdom; b Respiratory Medicine Unit, Nuffield Department of Medicine, University of Oxford, Oxford, United Kingdom; c Peter Medawar Building for Pathogen Research, Nuffield Department of Medicine, University of Oxford, Oxford, United Kingdom; d MRC Human Immunology Unit, Weatherall Institute of Molecular Medicine, John Radcliffe Hospital, University of Oxford, Oxford, United Kingdom; e Translational Gastroenterology Unit, Nuffield Department of Medicine, John Radcliffe Hospital, University of Oxford, Oxford, United Kingdom; f Atopix Therapeutics Limited, Abingdon, United Kingdom

**Keywords:** Prostaglandin D_2_, leukotriene E_4_, chemoattractant receptor-homologous molecule expressed on T_H_2 cells, T_H_2 cells, neutrophils, CAIA, Cell activation–induced aggregation, CRTH2, Chemoattractant receptor-homologous molecule expressed on T_H_2 cells, cysLT, Cysteinyl leukotriene, CysLT_1_, Cysteinyl leukotriene receptor 1, CysLT_2_, Cysteinyl leukotriene receptor 2, ICAM, Intercellular adhesion molecule, LTC_4_, Cysteinyl leukotriene C_4_, LTD_4_, Cysteinyl leukotriene D_4_, LTE_4_, Cysteinyl leukotriene E_4_, PGD_2_, Prostaglandin D_2_, PI3K, Phosphoinositide 3-kinase, PMA, Phorbol 12-myristate 13-acetate, qPCR, Quantitative PCR, RORγt, Retinoic acid–related orphan receptor γt

## Abstract

**Background:**

Prostaglandin D_2_ (PGD_2_) and cysteinyl leukotrienes (cysLTs) are lipid mediators derived from mast cells, which activate T_H_2 cells. The combination of PGD_2_ and cysLTs (notably cysteinyl leukotriene E_4_ [LTE_4_]) enhances T_H_2 cytokine production. However, the synergistic interaction of cysLTs with PGD_2_ in promoting T_H_2 cell activation is still poorly understood. The receptors for these mediators are drug targets in the treatment of allergic diseases, and hence understanding their interaction is likely to have clinical implications.

**Objective:**

We aimed to comprehensively define the roles of PGD_2_, LTE_4_, and their combination in activating human T_H_2 cells and how such activation might allow the T_H_2 cells to engage downstream effectors, such as neutrophils, which contribute to the pathology of allergic responses.

**Methods:**

The effects of PGD_2_, LTE_4_, and their combination on human T_H_2 cell gene expression were defined by using a microarray, and changes in specific inflammatory pathways were confirmed by means of PCR array, quantitative RT-PCR, ELISA, Luminex, flow cytometry, and functional assays, including analysis of downstream neutrophil activation. Blockade of PGD_2_ and LTE_4_ was tested by using TM30089, an antagonist of chemoattractant receptor-homologous molecule expressed on T_H_2 cells, and montelukast, an antagonist of cysteinyl leukotriene receptor 1.

**Results:**

PGD_2_ and LTE_4_ altered the transcription of a wide range of genes and induced diverse functional responses in T_H_2 cells, including cell adhesion, migration, and survival and cytokine production. The combination of these lipids synergistically or additively enhanced T_H_2 responses and, strikingly, induced marked production of diverse nonclassical T_H_2 inflammatory mediators, including IL-22, IL-8, and GM-CSF, at concentrations sufficient to affect neutrophil activation.

**Conclusions:**

PGD_2_ and LTE_4_ activate T_H_2 cells through different pathways but act synergistically to promote multiple downstream effector functions, including neutrophil migration and survival. Combined inhibition of both PGD_2_ and LTE_4_ pathways might provide an effective therapeutic strategy for allergic responses, particularly those involving interaction between T_H_2 cells and neutrophils, such as in patients with severe asthma.

Prostaglandin D_2_ (PGD_2_) and cysteinyl leukotrienes (cysLTs) are lipid mediators released from mast cells.^1^ Both are detected in high concentrations at sites of allergic inflammation, playing critical roles in the pathogenesis of allergic disorders.^2,3^

A significant contribution of PGD_2_ to the development of allergic inflammation has been suggested by the observation of enhanced eosinophilic lung inflammation and cytokine release in transgenic mice overexpressing PGD_2_ synthase.^4^ Two PGD_2_ receptors have been identified: D prostanoid receptor 1 and chemoattractant receptor-homologous molecule expressed on T_H_2 cells (CRTH2).^5^ CRTH2 is highly expressed in human eosinophils, basophils, T_H_2 cells, and group 2 innate lymphoid cells.^6-8^ Evidence suggests that the proinflammatory role of PGD_2_ in these cells is predominantly mediated by CRTH2. Through CRTH2, PGD_2_ elicits chemotaxis,^6,7,9^ stimulates type 2 cytokine production,^9-11^ and suppresses apoptosis.^12^

CysLTs, including cysteinyl leukotriene C_4_ (LTC_4_), cysteinyl leukotriene D_4_ (LTD_4_), and cysteinyl leukotriene E_4_ (LTE_4_), are derived from the 5-lipoxygenase pathway of arachidonic acid metabolism. Two G protein–coupled receptors for cysLTs have been characterized and designated as cysteinyl leukotriene receptor 1 (CysLT_1_) and cysteinyl leukotriene receptor 2 (CysLT_2_).^13,14^ CysLT_1_ mediates bronchoconstriction and proinflammatory effects, including activation and migration of leukocytes.^15,16^ CysLT_1_ antagonists, including montelukast, are approved for clinical use in patients with asthma and allergic rhinitis.

We reported recently that cysLTs potentiated type 2 cytokine production from human T_H_2 cells in response to PGD_2_.^17^ The combination of a CRTH2 antagonist and montelukast was required to completely inhibit type 2 cytokine production induced by mast cell supernatants. These data highlighted an interaction between PGD_2_ and cysLTs in promoting mast cell–mediated T_H_2 cell activation. To date, understanding of the synergistic effects of these lipids on T_H_2 cell function is limited to type 2 cytokine production. Hence we investigated their effects on additional mediators of allergic inflammation and their roles in triggering diverse T_H_2 cell responses. In particular, we addressed their ability to crosstalk with neutrophils, which are critical players in allergic inflammation, particularly in patients with severe asthma.^18,19^ Because our previous studies had identified LTE_4_ as the most potent cysLT in T_H_2 cytokine production,^17^ we focused on the effects of combining PGD_2_ and LTE_4_.

Our data demonstrate that the proinflammatory effects of both PGD_2_ and LTE_4_ in human T_H_2 cells reach far beyond type 2 cytokine production. Indeed, we find that these lipids synergistically upregulated expression of a range of genes associated with inflammation and confirm that this gene regulation enhances T_H_2 cell adhesiveness, migration, and survival and promotion of T_H_2 crosstalk with neutrophils *in vitro*. Hence we suggest that the synergistic action of PGD_2_ and LTE_4_ could contribute to neutrophilia in patients with severe asthma by inducing neutrophil chemokine and growth factor production by T_H_2 cells.

## Methods

### T_H_2 lymphocytes

**Fig E1:**
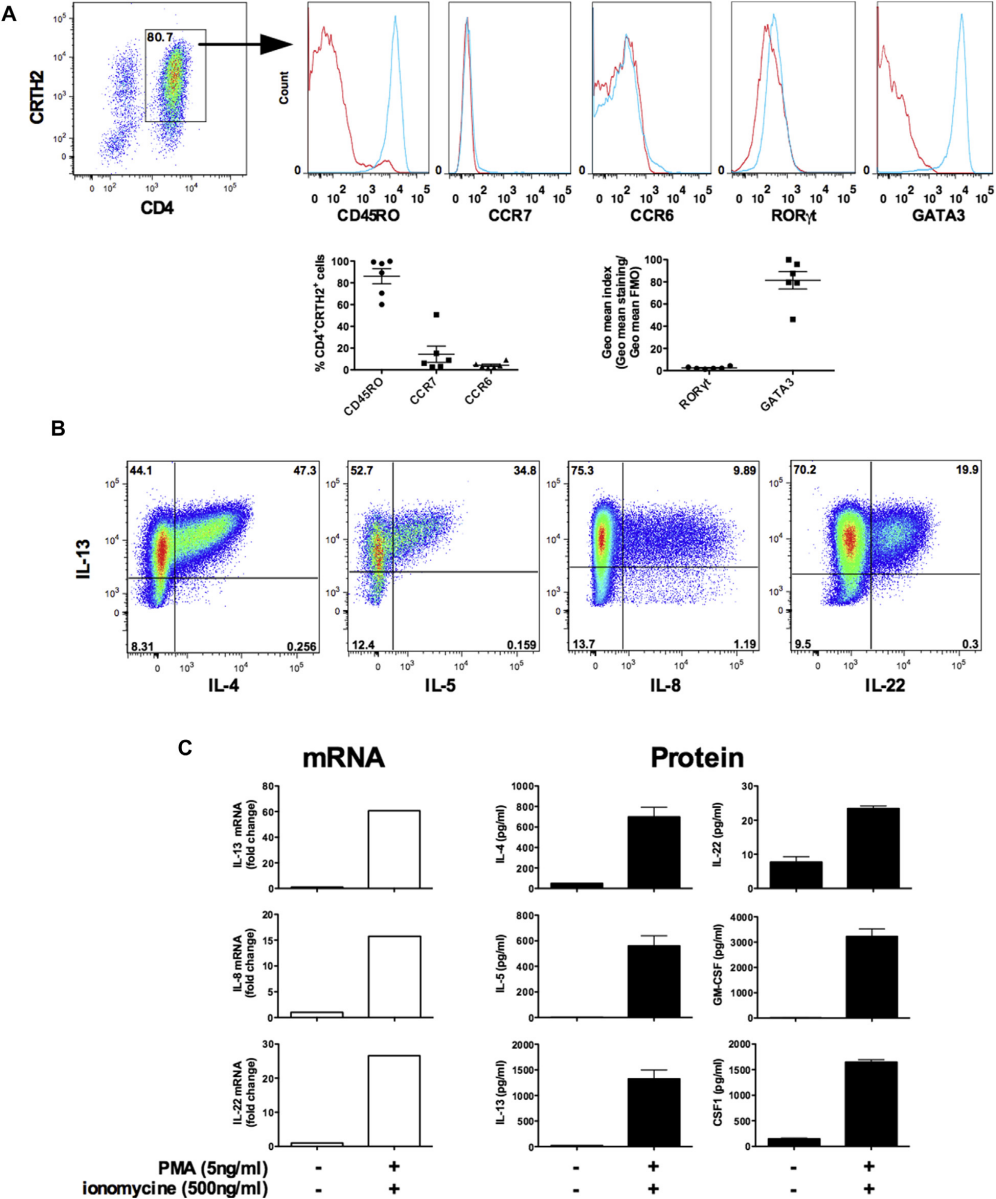
Analysis of cell phenotype. **A** and **B,** Expanded T_H_2 cells were CD3^+^CD4^+^CRTH2^+^CD45RO^+^GATA3^+^CCR6^−^CCR7^−^CD45RA^−^RORγt^−^ effector memory cells (Fig E1, *A*), which produce the type II cytokines IL-8 and IL-22 after stimulation with PMA (5 ng/mL) and ionomycin (500 ng/mL; Fig E1, *B*), as detected by means of flow cytometry. *Blue lines* represent staining of indicated cell markers, and *red lines* represent unstained controls. **C,** The same cytokine profile was observed in freshly isolated *ex vivo* T_H_2 cells by using qPCR (mRNA) and Luminex (protein) assays (high background IL-8 levels in the unstimulated sample precluded accurate analysis of this cytokine in the Luminex assay; n = 6 for Fig E1, *A* and *B*; n = 2 for mRNA and n = 1 for protein in Fig E1, *C*).

T_H_2 cells were isolated from buffy coats (National Blood Service, Bristol, United Kingdom), as described in the Methods section in this article's Online Repository at www.jacionline.org.^11^ They are memory cells showing a CD4^+^CRTH2^+^CD45RO^+^GATA3^+^CCR6^−^CD45RA^−^ retinoic acid–related orphan receptor γt (RORγt)^−^ phenotype with relatively high purity (see Fig E1, *A*, in this article's Online Repository at www.jacionline.org).

For analysis of gene regulation and cytokine production, T_H_2 cells were treated with PGD_2_ or LTE_4_ alone or their combination in X-VIVO 15 medium (Lonza, Basel, Switzerland) in the presence or absence of antagonist compounds for 2.5 hours (microarray, PCR array, and quantitative PCR [qPCR]) or 4 hours (Luminex).

Cells were treated with the same compounds in serum-free RPMI medium for 4 hours to prepare T_H_2 cell–conditioned media for neutrophil assays.

### Neutrophils

Human neutrophils were isolated from fresh whole blood. Briefly, the red blood cell pellet was collected after Ficoll-Paque Plus density gradient, suspended in HBSS, and mixed with 3% dextran. Neutrophil-rich supernatant was collected and treated in a 0.2% NaCl solution for red blood cell lysis and resuspended in RPMI medium.

### Microarrays

Total cellular RNA was extracted with RNeasy Mini kits (Qiagen, Hilden, Germany). Microarrays were performed by Cambridge Genomic Services (Cambridge, United Kingdom) using a HumanHT-12 v4 chip. Genes significant at a *P* value of less than .05 were analyzed by using the Venn Diagram module within GenePattern.^20^ Pathway analyses were conducted with IPA (Ingenuity Systems, www.ingenuity.com). Heat maps were generated by using GENE-E software (http://www.broadinstitute.org/cancer/software/GENE-E/index.html).

### PCR arrays

PCR arrays were performed with an RT^2^ Profiler PCR Array Human Common Cytokines kits (SABiosciences, Frederick, Md) in a LightCycler 480 Real-Time PCR System (Roche, Mannheim, Germany).

### Luminex

Cytokines were measured with a Procarta Human Cytokine Immunoassay kit (Affymetrix, Santa Clara, Calif). The results were obtained with a Bio-Plex 200 System (Bio-Rad Laboratories, Hercules, Calif).

### qPCR

qPCR was performed, as described previously.^17^ Primers and probes (Roche) used are listed in Table E1 in this article's Online Repository at www.jacionline.org.

### ELISA

Cytokines were assayed with ELISA kits (R&D Systems, Minneapolis, Minn). The results were measured in a FLUOstar OPTIMA luminescence plate reader (BMG LabTech, Ortenberg, Germany).

### Flow cytometric analysis

Cells were labeled with antibody to CD16–fluorescein isothiocyanate or Annexin V–allophycocyanin and then acquired with an LSR II Flow Cytometer (BD Biosciences, San Jose, Calif).

### Cell aggregation analysis

Cell aggregation was photographed with a Nikon Eclipse TS100 microscope (Nikon, Tokyo, Japan). Images were analyzed with CellProfiler 2 software (Broad Institute, Cambridge, Mass; also see the Methods section in this article's Online Repository).^21^

### Chemotaxis assays

Chemotaxis assays were conducted, as described previously.^9^

### Statistics

Data were analyzed by using 1-way ANOVA, followed by the Newman-Keuls test. *P* values of less than .05 were considered statistically significant.

## Results

### Effect of PGD_2_ and LTE_4_ on the gene expression profile of T_H_2 cells

PGD_2_ and LTE_4_ synergistically evoke type 2 cytokine production from human T_H_2 cells.^17^ To understand their broader synergistic effects on T_H_2 cell function, we investigated the transcriptional responses to LTE_4_ or PGD_2_ added either alone or in combination by using RNA microarrays. Three experimental replicates were prepared for each of the 4 groups (control, LTE_4_, PGD_2_, and their combination). The concentrations of LTE_4_ (50 nmol/L) and PGD_2_ (100 nmol/L) used for the treatments were close to their relative median effective concentration values for type 2 cytokine production in T_H_2 cells.^17^

**Fig 1:**
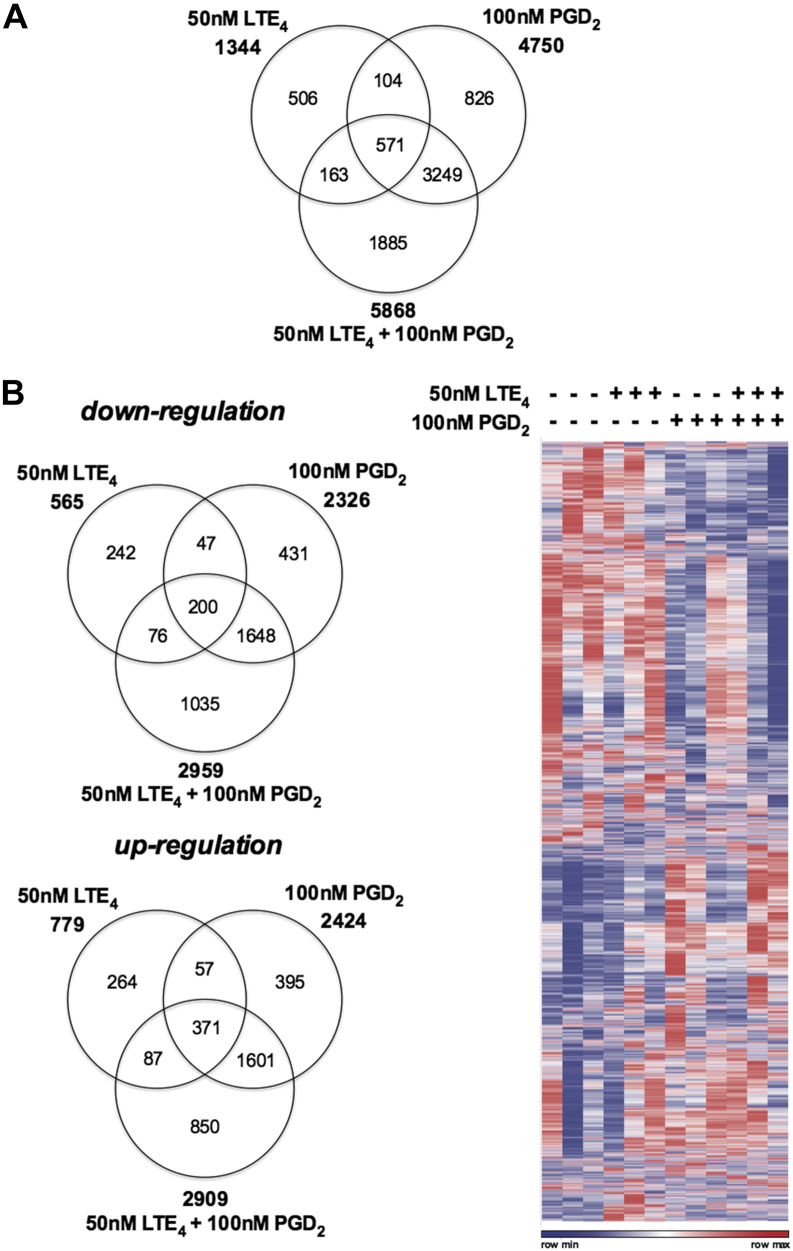
Gene regulation in T_H_2 cells by PGD_2_ and LTE_4_ detected by using a microarray. **A,** Venn diagram representing total numbers of genes regulated significantly. **B,** Venn diagrams and heat map showing numbers of genes downregulated or upregulated significantly. *P* < .05.

The data showed broad transcriptional changes after treatment. The mRNA levels of 1344, 4750, and 5868 genes were significantly (*P* < .05) modulated (including upregulation and downregulation) by LTE_4_, PGD_2_, or their combination, respectively (Fig 1, *A*). The effect of PGD_2_ was much broader than that of LTE_4_. Although some (approximately 675) of the gene responses overlapped, most of them were regulated distinctly: 669 only by LTE_4_ and 4075 only by PGD_2_. The combination of LTE_4_ and PGD_2_ amplified significantly the range of the transcriptional response. Expression of a group of 1885 genes was altered only by combination treatment, indicating the combinatorial effect of PGD_2_ and LTE_4_ on gene expression. Among the modulated genes, about half were upregulated and half were downregulated (Fig 1, *B*).

The genes regulated included those involved in the pathways critical for T-cell intrinsic functions and interactions with other cell types (through cell-surface receptors and secreted mediators). To define the significance of the gene expression changes specifically in relation to T_H_2-mediated allergic inflammation, we analyzed first the effect of lipid mediators on T-cell intrinsic functions and then the effect on cytokine-driven crosstalk with downstream effector cells.

### Effects of PGD_2_ and LTE_4_ on the apoptosis and migration of T_H_2 cells

**Fig E2:**
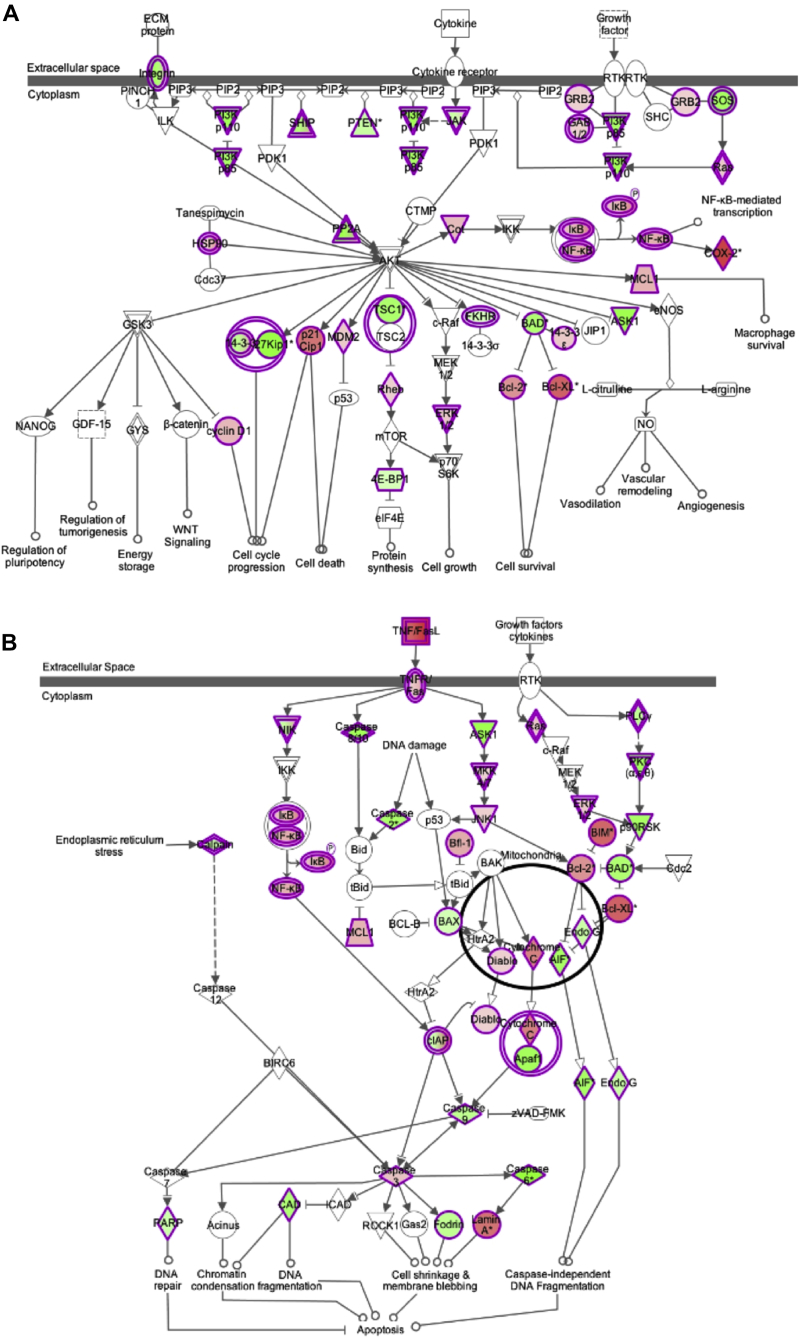
Network diagram of genes depicting pathways involved in activation of T_H_2 cells induced by PGD_2_ and LTE_4_ based on microarray data. **A,** PI3K pathway. **B,** Apoptosis pathway. *Red color* shows gene upregulation, and *green color* shows downregulation.

**Fig E3:**
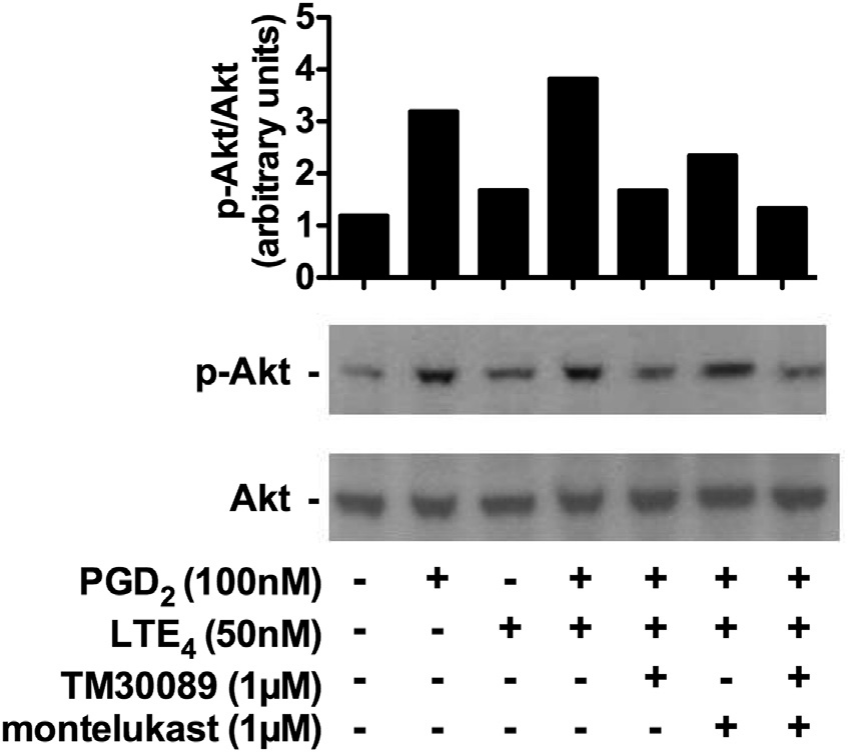
Phosphorylation of Akt in T_H_2 cells after treatment with PGD_2_ and LTE_4_ in the presence or absence of TM30089 and montelukast. The intensity of the bands for phospho-Akt was quantified after normalization with the bands for total Akt.

**Fig 2:**
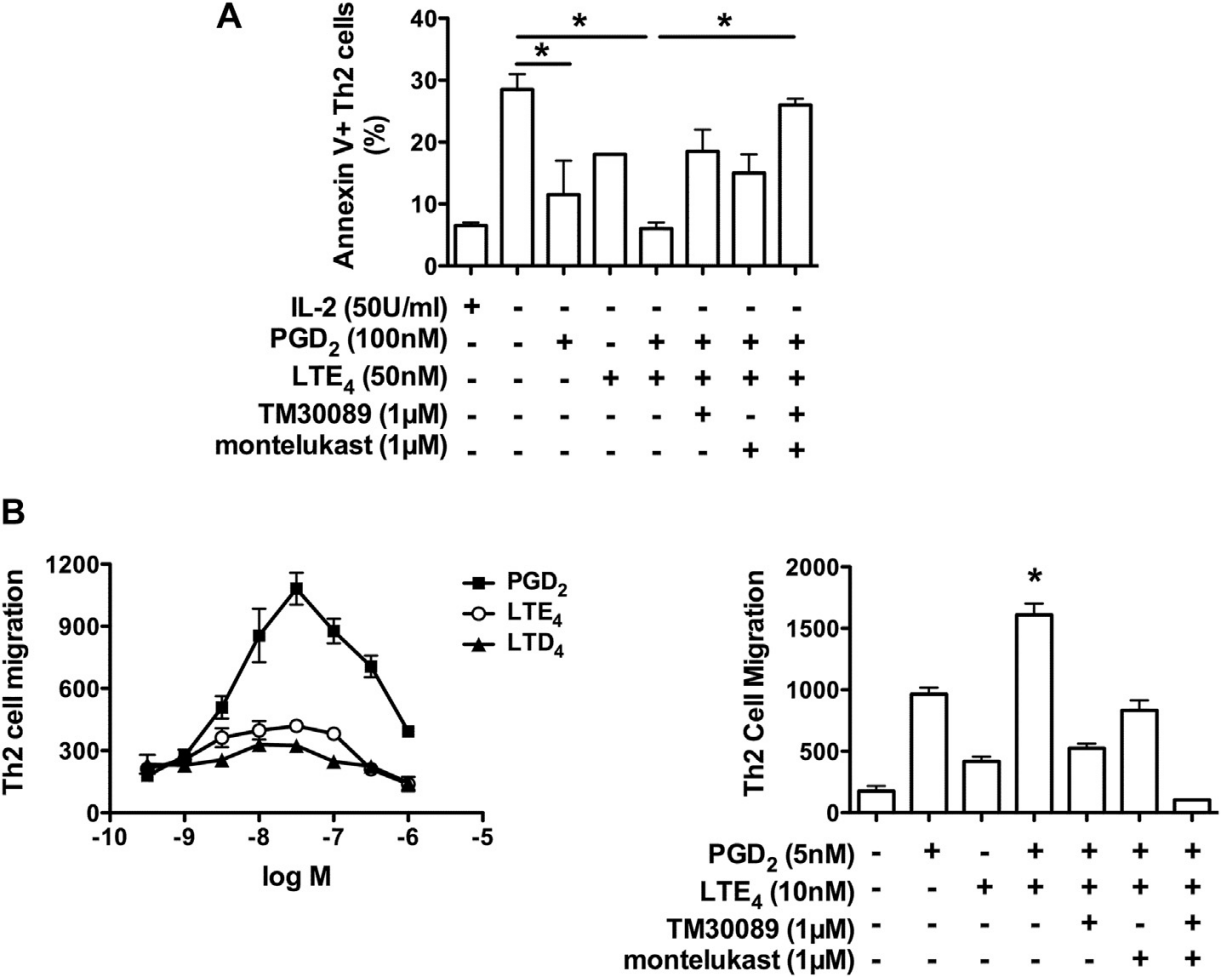
Effects of PGD_2_ and LTE_4_ on the apoptosis and migration of T_H_2 cells. **A,** Detection of Annexin V in T_H_2 cells treated with IL-2 deprivation in the presence of compounds, as indicated. **B,** Cell migration in response to PGD_2_, LTE_4_, or LTD_4_*(left panel)* or the combination of indicated compounds *(right panel)*. *P < .05 between indicated treatments (Fig 2, *A*) or between PGD_2_ plus LTE_4_ and other treatments (Fig 2, *B*). n = 3.

Ingenuity pathway analysis of the microarray data suggested that PGD_2_ and LTE_4_ treatment altered the expression of clusters of genes associated with distinct cell-signaling pathways in T_H_2 cells, including the phosphoinositide 3-kinase (PI3K) and apoptosis pathways (see Fig E2 in this article's Online Repository at www.jacionline.org). Western blotting for phospho-Akt also confirmed that activation of the PI3K pathway by PGD_2_ and LTE_4_ was inhibited by TM30089 and montelukast (see Fig E3 in this article's Online Repository at www.jacionline.org). PI3K pathway signaling is critical in mediating the antiapoptotic and chemotactic roles of PGD_2_/CRTH2.^12,22^ Therefore we further addressed the effect of PGD_2_, LTE_4_, and their combination on these functions (Fig 2).

In the case of apoptosis, both PGD_2_ and LTE_4_ markedly reduced upregulation of Annexin V in T_H_2 cells after 16 hours of IL-2 withdrawal (Fig 2, *A*). The combination of 2 mediators additively enhanced this effect.

In chemotaxis assays we first compared the chemotactic effect of PGD_2_ with that of cysLTs, including LTD_4_ and LTE_4_ (Fig 2, *B*, left panel). These induced migration in a dose-dependent manner, peaking around 30 nmol/L for PGD_2_ and LTE_4_ and 20 nmol/L for LTD_4_. The maximum response achieved by LTE_4_ was higher (approximately 2-fold) than that elicited by LTD_4_ but only approximately 27% of that elicited by PGD_2_. The combination of PGD_2_ and LTE_4_ at concentrations close to their median effective concentration synergistically enhanced cell migration (Fig 2, *B*, right panel).

In both apoptosis and chemotaxis assays the contribution of PGD_2_ and LTE_4_ was ablated by TM30089 and montelukast, respectively, and the combination of TM30089 and montelukast inhibited almost all cell responses induced by the combination of PGD_2_ and LTE_4_.

### Effects of PGD_2_ and LTE_4_ on expression of adhesion molecules in T_H_2 cells

Inspection of the microarray data indicated induction by PGD_2_ and LTE_4_ of a particularly large number of transcripts associated with leukocyte adhesion (Table I). Prominent among these were the integrins αV *(ITGAV)*, α2 *(ITGA2)*, αE *(ITGAE)*, and α11 *(ITGA11)*, which constitute subunits of the leukocyte receptors α2βL for intercellular adhesion molecule 1 (ICAM-1) on endothelium, αEβ7 for E-cadherins on epithelium, and α2β/αVβ3 for collagen and vitronectin in tissue extracellular matrix. In addition, PGD_2_ and LTE_4_ induced expression of transcripts encoding ICAM-1 *(ICAM1)* and ICAM-2 *(ICAM2)*, as well as the homophilic adhesion molecule CD31 *(PECAM1)* and the cadherin/catenin protein family members *CTNNAL1*, *CTNNA1*, *CTNND1*, *PCDHA1*, *PCDHA4*, and *CDH1*, which also act as homophilic adhesion receptors. The majority of these genes (n = 14) were induced by PGD_2_ alone, whereas only 3 (*ITGAV*, *CTNND1*, and *ITGA11*) were induced by LTE_4_. Upregulation of some genes (*IGSF3*, *CTNNA1*, *NINJ1*, *DCHS1*, *PCDHA4*, and *ITGA2*) was amplified by the combination of PGD_2_ and LTE_4_.

**Fig 3:**
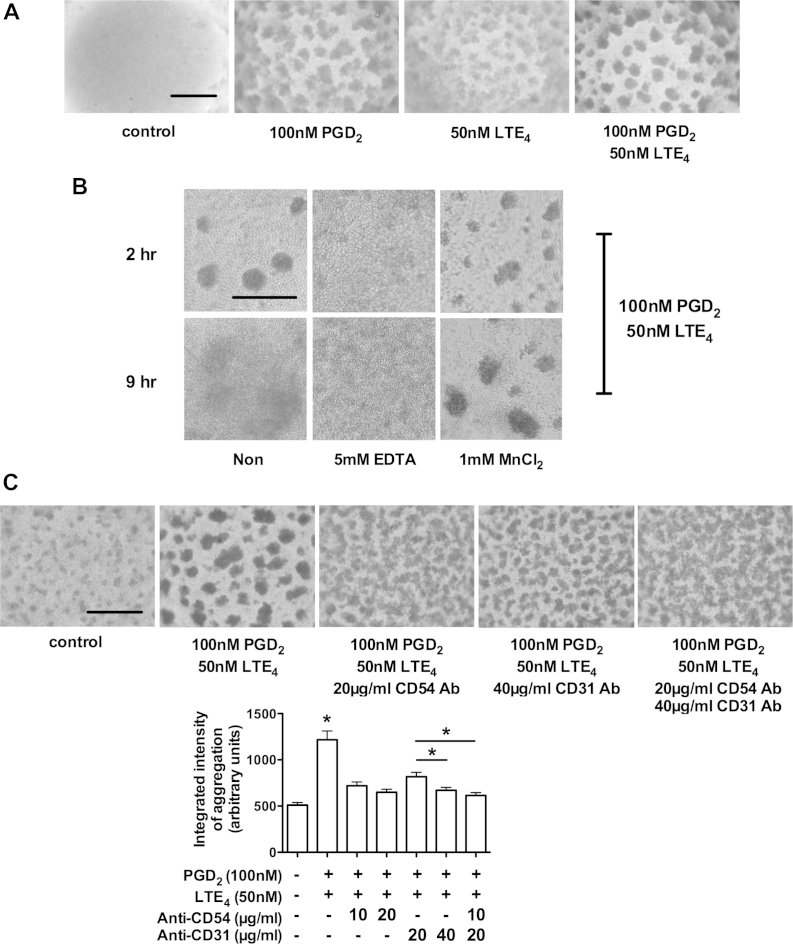
Involvement of integrins in CAIA of T_H_2 cells induced by PGD_2_ and LTE_4_. Cell aggregation after incubation with indicated treatments for 2 hours **(A)**, for 2 or 9 hours in the presence of EDTA or MnCl_2_**(B)**, or for 1 hour in the presence of the indicated antibodies **(C)**. *Scale bar* = 0.5 mm. *P < .05 between PGD_2_ plus LTE_4_ and other treatments or between indicated conditions (n = 2-5).

To explore the consequences of such increased integrin expression on T_H_2 cell-cell adhesion, we used an *in vitro* cell activation–induced aggregation (CAIA) assay.^23^ Stimulation with either lipid alone caused marked CAIA that formed within 0.5 and 1 hours and persisted for 4 to 6 hours (Fig 3, *A*). The combination of both mediators further enhanced the aggregation. To verify the involvement of integrins, which are critically dependent on Ca^2+^ ions, we tested the effect of EDTA, an inhibitory chelating agent, and MnCl_2_, an activator of integrin function, on PGD_2_/LTE_4_-induced CAIA. As shown in Fig 3, *B*, EDTA (5 mmol/L) inhibited and MnCl_2_ (1 mmol/L) prolonged the CAIA. Blocking antibodies to CD54 *(ICAM1)* and CD31 *(PECAM1)* were used to further confirm the contribution of integrins to the CAIA (Fig 3, *C*). Both antibodies partially reduced the intensity of CAIA in a concentration-dependent manner. The inhibitory effect of anti-CD54 was slightly more potent than that of anti-CD31, and the combination showed a marginal additive effect.

### Enhancement of proinflammatory protein production by PGD_2_ and LTE_4_ in T_H_2 cells

**Fig 4:**
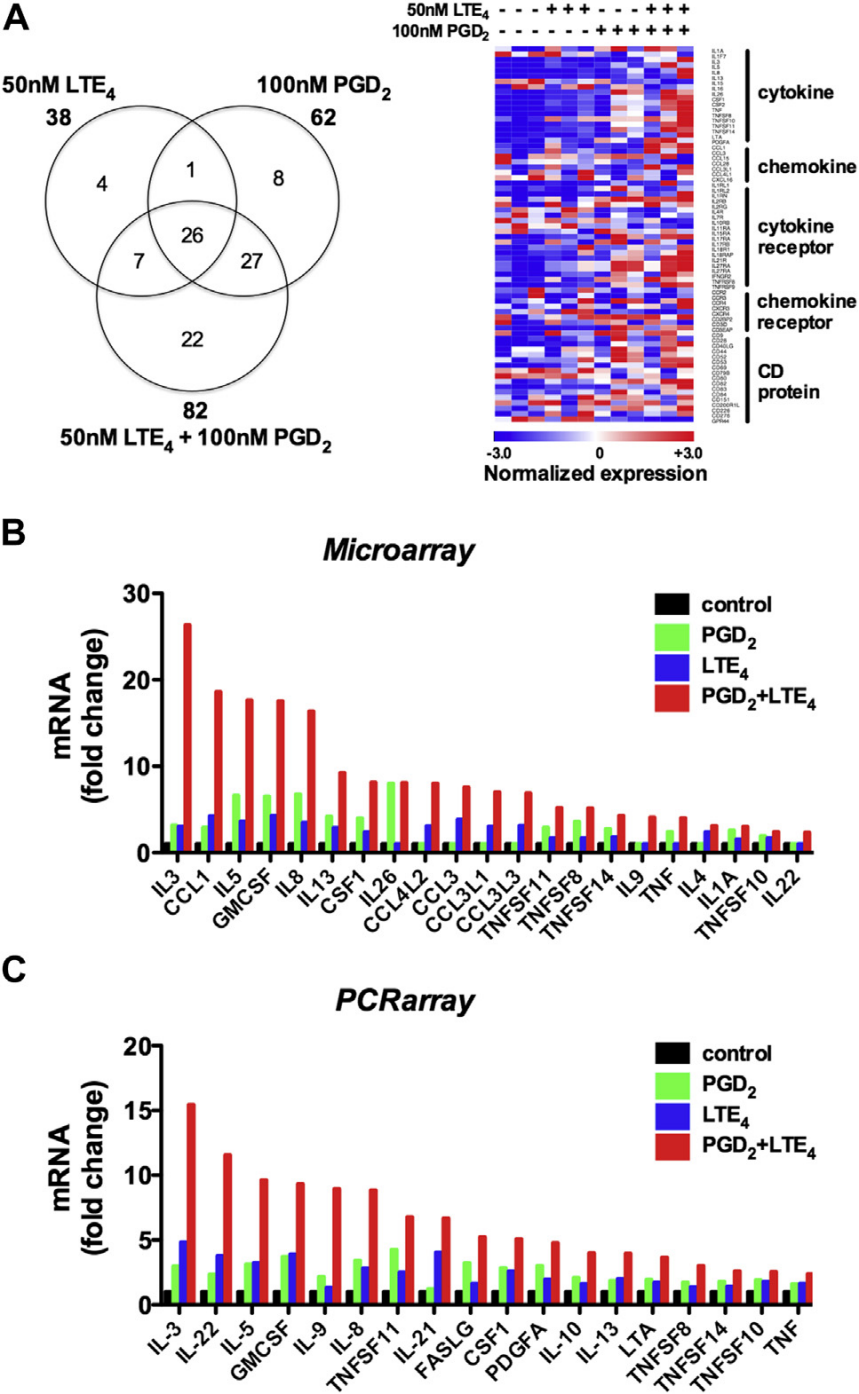
PGD_2_ and LTE_4_ modulate gene transcription of cytokines, chemokines, and surface receptors in T_H_2 cells determined by means of microarray (**A** and **B**) or PCR array **(C)**. Fig 4, *A*, Venn diagram and heat map showing the number and distribution of genes significantly regulated. Fig 4, *B* and *C*, Strongly upregulated genes. *P* < .05 (n = 3).

To focus on gene regulation potentially relevant to T cell–mediated diseases, we next analyzed the genes encoding cytokines, chemokines, their receptors, and cluster of differentiation (CD) molecules detected by the microarray (Fig 4). A total of 95 of these genes were significantly modulated, most of them upregulated (Fig 4, *A*, and see Table E2 in this article's Online Repository at www.jacionline.org), most significantly the cytokines and chemokines. Although some of these effects were induced by PGD_2_ alone (eg, *IL26*, *IL1RL1*, and *CCR4*) or LTE_4_ alone (eg, *CCL3*, *CCL3L1*, *CCL3L3*, and *CCL4L2*), most were driven by their combination. In addition to type 2 cytokine genes, the expression of many other genes was also synergistically enhanced by the combination treatment (Fig 4, *B*). A number of genes were downregulated (see Table E2), notably transcription of *GPR44*, the gene for CRTH2 (CD294), which was downregulated by 2.4-fold by PGD_2_ alone and 3.8-fold by combination treatment. Importantly, these microarray data were largely confirmed also by using a PCR array assay for human common cytokines, including 84 cytokine genes, among which approximately 30 showed significant changes (see Table E3 in this article's Online Repository at www.jacionline.org), although some effects (*IL10* and *IL21*) were only detected by using the PCR array (Fig 4, *C*).

**Fig E4:**
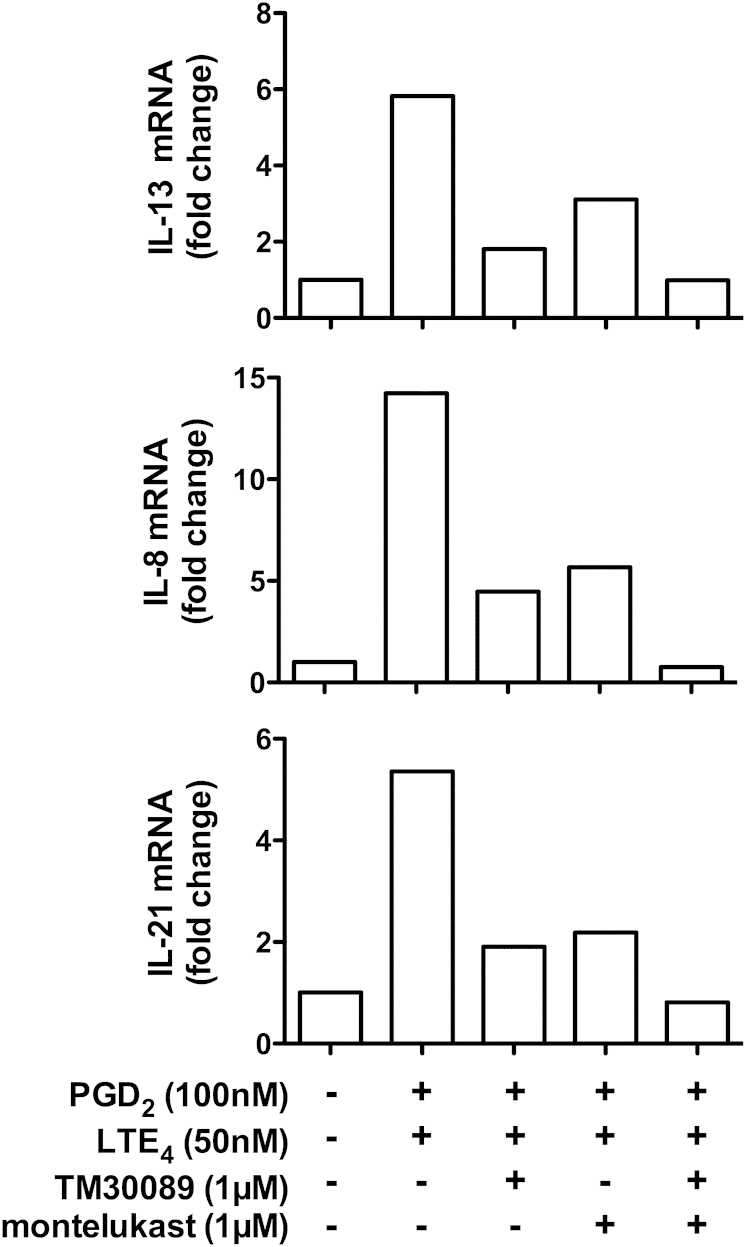
Effect of TM30089 and montelukast on transcriptional regulation of cytokine genes induced by PGD_2_ and LTE_4_ in T_H_2 cells determined by using qPCR. The control sample was treated as 1-fold (n = 1).

**Fig 5:**
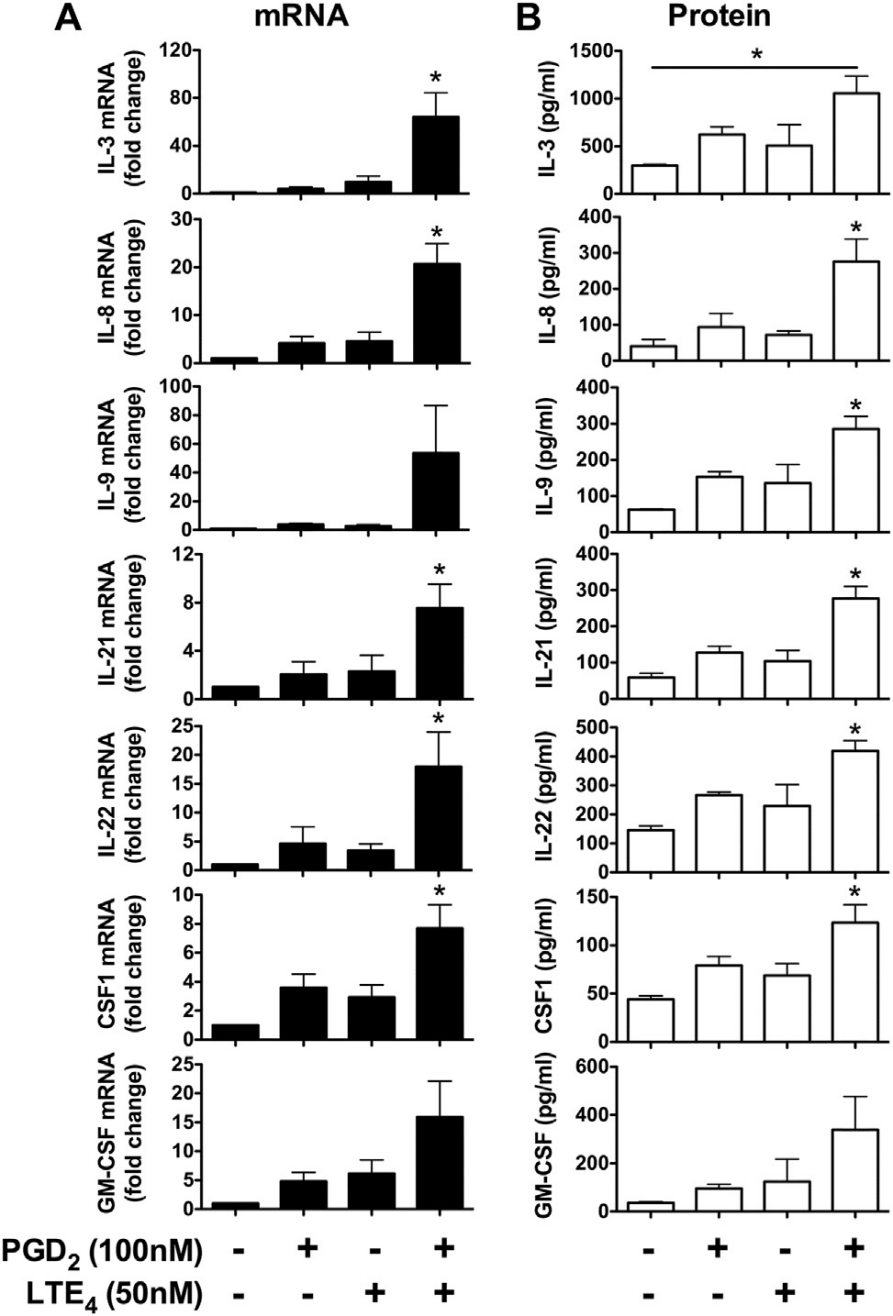
Effects of PGD_2_ and LTE_4_ on production of selected cytokines in T_H_2 cells. **A,** Levels of mRNA measured by using qPCR. The mRNA levels in control samples were treated as 1-fold. **B,** Protein levels were detected with the Luminex assay. *P < .05 between PGD_2_ plus LTE_4_ and other conditions or the indicated condition (n = 3).

To further verify our findings, we also conducted qPCR (Fig 5, *A*) and Luminex (Fig 5, *B*) assays on selected cytokines. The qPCR data strongly supported the synergistic effects of PGD_2_ and LTE_4_. At the protein level, combination treatment either additively (IL-3, IL-22, and macrophage colony-stimulating factor) or synergistically (IL-8, IL-9, IL-21, and GM-CSF) enhanced the cytokine production. A similar cytokine profile was also observed after stimulation with phorbol 12-myristate 13-acetate (PMA)/ionomycin in both cultured T_H_2 cells (by means of intracellular cytokine staining) and *ex vivo* T_H_2 cells (by means of qPCR/Luminex; see Fig E1, *B* and *C*). As expected, TM30089 or montelukast only partially inhibited cytokine upregulation after PGD_2_ plus LTE_4_, but a combination of TM30089 and montelukast completely blocked this effect (see Fig E4 in this article's Online Repository at www.jacionline.org).

### Effect of cytokines induced by PGD_2_ and LTE_4_ from T_H_2 cells on neutrophil function

**Fig 6:**
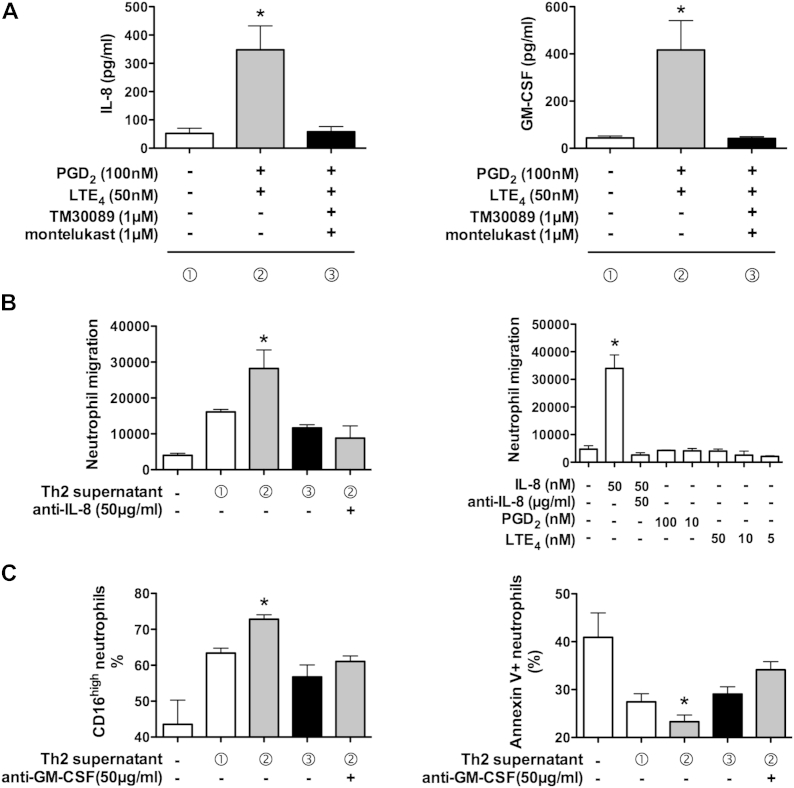
T_H_2-derived cytokines activate neutrophils. **A,** IL-8 and GM-CSF levels in stimulated T_H_2 cell supernatants assigned as supernatants 1 *(white bars)*, 2 *(gray bars)*, and 3 (*black bars*, n = 4). **B,** Effect of supernatants *(left panel)*, IL-8, PGD_2_, LTE_4_*(right panel)*, and anti–IL-8 antibody on neutrophil migration. **C,** Effect of supernatants and anti–GM-CSF antibody on expression of CD16 *(left panel)* and Annexin V *(right panel)* in neutrophils determined by using fluorescence-activated cell sorting. *P < .05 between the indicated treatment and other conditions (n = 2-7).

Neutrophilia is detected in the majority of patients with severe asthma,^19^ and the cytokines induced by activation of T_H_2 cells included several that could potentially interact with neutrophils. To explore this possibility, we tested the ability of IL-8 and GM-CSF produced by T_H_2 cells to elicit relevant changes in human neutrophil behavior (Fig 6). As indicated by the data in Fig 6, *A*, treatment with PGD_2_ and LTE_4_ promoted secretion of IL-8 and GM-CSF from resting levels (<90 and <70 pg/mL, respectively, in supernatant 1 to more than 560 and 650 pg/mL, respectively, in supernatant 2; Fig 6, *A*). This was inhibited by coincubation with TM30089 and montelukast (approximately 94 pg/mL IL-8 and approximately 50 pg/mL GM-CSF in supernatant 3).

**Fig E5:**
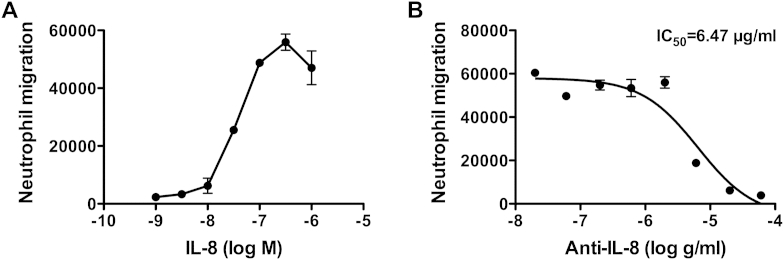
Effect of IL-8 on neutrophil migration. **A,** Neutrophil migration to various concentrations of rhIL-8 in chemotaxis assay. **B,** Effect of increasing concentration of anti–IL-8 antibody on neutrophil migration induced by 50 nmol/L rhIL-8.

First, we addressed the effect of endogenous IL-8 on neutrophil chemotaxis. Recombinant IL-8 induced neutrophil migration with a typical chemotaxis dose curve (see Fig E5, *A*, in this article's Online Repository at www.jacionline.org). This effect could be inhibited by a neutralizing antibody against IL-8 in a dose-dependent manner (see Fig E5, *B*). The T_H_2 supernatant containing high levels of IL-8 (supernatant 2) had a strong capacity to induce neutrophil migration (Fig 6, *B*, left panel). Inhibition of IL-8 production by TM30089 and montelukast caused substantial (approximately 43%) reduction of the chemotactic activity of supernatant 3. The neutrophil migration to supernatant 2 was mostly blocked by IL-8 neutralizing antibody. To rule out the possibility that cell migration was induced directly by PGD_2_ or LTE_4_ (used to prepare supernatant 2), we examined the effects of these mediators (Fig 6, *B*, right panel). Neither was chemotactic for neutrophils.

**Fig E6:**
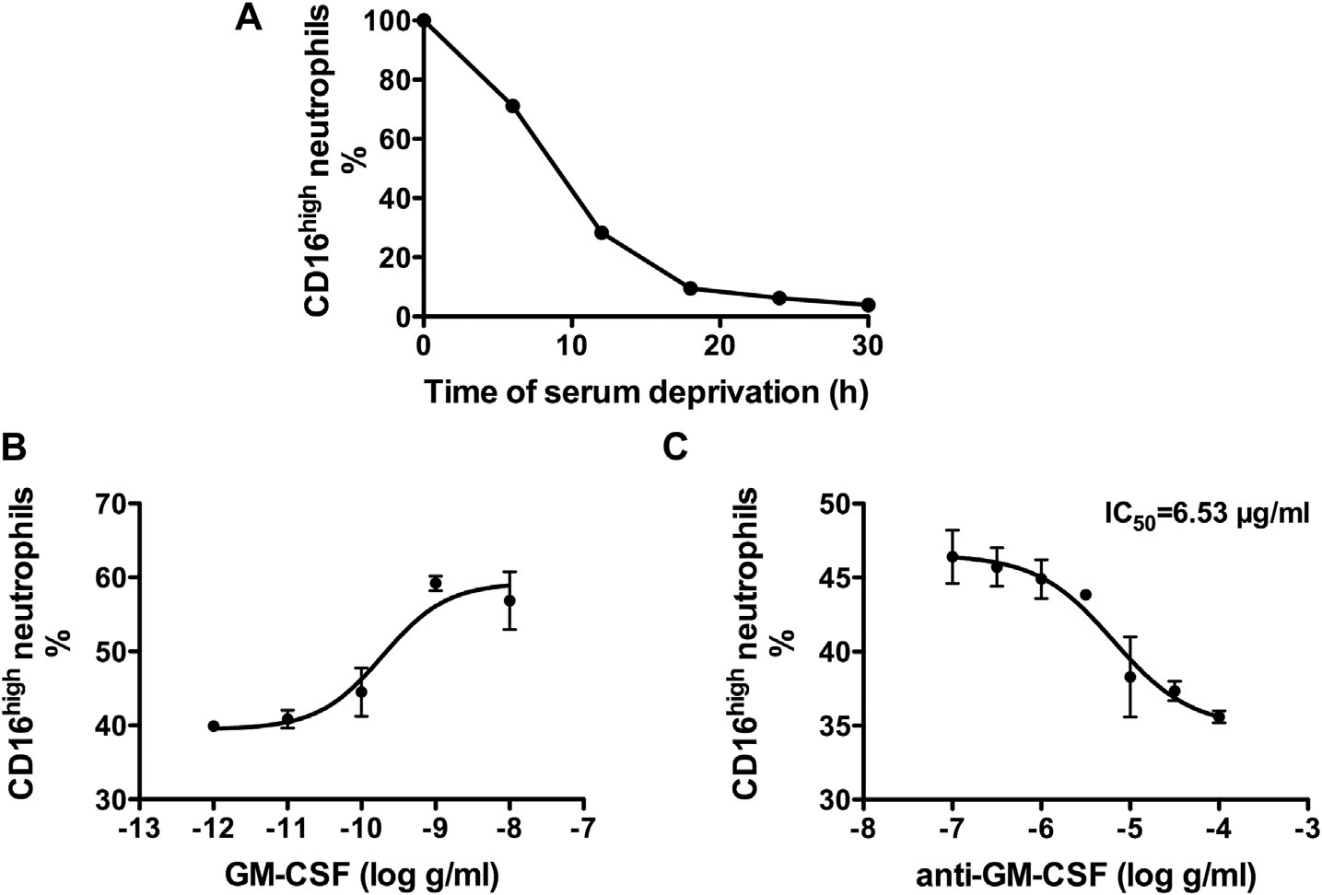
Effect of GM-CSF on the decrease in CD16 levels in neutrophils, a biomarker of apoptosis, induced by serum deprivation. **A,** Decrease of CD16^high^ neutrophils with the time of serum deprivation. **B,** Inhibitory effect of various concentration of rhGM-CSF on the decrease of CD16^high^ neutrophils induced by serum deprivation for 12 hours. **C,** The inhibitory effect of rhGM-CSF (1 ng/mL) was reversed by anti–GM-CSF antibody in a dose-dependent manner. *IC*_*50*_, Inhibitory concentration of 50%.

Next, we studied the influence of endogenous GM-CSF on neutrophil behavior by measuring its ability to rescue cells from serum starvation–induced apoptosis using CD16 (FcγRIII) expression as a biomarker of cell integrity (see Fig E6, *A*, in this article's Online Repository at www.jacionline.org).^24^ Confirming the validity of the assay, the numbers of CD16^high^ neutrophils decreased after serum withdrawal, which was inhibited by recombinant GM-CSF in a dose-dependent manner (see Fig E6, *B*). The inhibitory effect of GM-CSF was reversed by a neutralizing antibody against human GM-CSF (see Fig E6, *C*). Importantly, similar protection against apoptosis was observed when T_H_2 cell supernatants, particularly supernatant 2, were substituted for recombinant GM-CSF (Fig 6, *C*, left panel). The protective capacity depended on the level of GM-CSF in the supernatant, which was reduced either by the inhibition of GM-CSF production in T_H_2 culture (supernatant 3) or GM-CSF neutralizing antibody. To further confirm the antiapoptotic activity of the endogenous GM-CSF induced by PGD_2_ and LTE_4_, we also examined the expression of Annexin V in neutrophils (Fig 6, *C*, right panel). As expected, T_H_2 supernatants, especially supernatant 2, reduced the numbers of Annexin V–positive cells markedly, which was partially inhibited by the GM-CSF antibody.

## Discussion

T_H_2 cells play an important role in type II immunity, particularly in mast cell–mediated allergic responses, by releasing high levels of type 2 cytokines.^25^ Our previous study demonstrated that PGD_2_ and cysLTs are the dominant mediators from activated mast cells that induce T_H_2 cytokine production.^17^ A combination of these mediators, particularly PGD_2_ and LTE_4_, synergistically enhances this response. In this study we further explored the role of PGD_2_ and LTE_4_ and their synergistic effect on diverse T_H_2 cell functions and revealed that the proinflammatory effects of these mediators are broader than previously recognized. Through activation of CRTH2 and a montelukast-sensitive receptor, gene expression was widely regulated; a number of cytokines, chemokines, and adhesion molecules were upregulated; and several cell-signaling pathways associated with cell adhesion and migration and apoptosis were activated. The upregulated cytokines and other proteins were functional to amplify proinflammatory responses of both T_H_2 cells themselves and downstream effectors. Combinations of PGD_2_ and LTE_4_ showed synergistic effects on these responses. Our findings provide novel insight into the critical role of PGD_2_ and LTE_4_, which contribute to important aspects of mast cell/T_H_2 cell–mediated allergic disorders.

Both PGD_2_ and LTE_4_ are lipid mediators involved in a wide range of chronic inflammatory disorders, including allergic asthma and rhinitis.^26,27^ Bronchoalveolar lavage fluid PGD_2_ and urinary LTE_4_ levels are increased in asthmatic patients. The role of PGD_2_ has been well studied, but the role of LTE_4_ and the molecular mechanisms used by these mediators are still obscure.^10-12^ For the first time, we show microarray analysis of the effects of these mediators in human T_H_2 cells, which suggested that their effects are not limited to type 2 cytokines but rather include a broad range of different genes. In general, the responses to PGD_2_ were more pronounced than those to LTE_4_. Although many genes are regulated by both lipids, these lipids seem to use different signaling mechanisms because most genes detected in the microarray were regulated distinctly. However, these mechanisms interact with each other because the combination of the lipids enhances gene regulation through both the intensity and number of genes. Although it has been reported that LTE_4_ activates the extracellular signal-regulated kinase pathway in human mast cells,^28^ no phosphorylation of extracellular signal-regulated kinase was detected in PGD_2_-activated T_H_2 cells.^24^ Intriguingly, the microarray data in this study indicate that the PI3K pathway is involved in both responses. Therefore further studies will be required to understand how the signals from these lipids interact to synergistically amplify their proinflammatory effects.

It is well established that activation of T_H_2 cells is characterized by production of high levels of type 2 cytokines that in turn promote type 2 responses in patients with allergic diseases. A subset of T_H_2 cells (CD4^+^CRTH2^+^CCR6^+^RORγt^+^) can also produce IL-17.^29^ In this study we demonstrated that T_H_2 cells activated by PGD_2_/LTE_4_ and other stimulations, such as PMA/ionomycin, could produce many other proinflammatory cytokines and chemokines that could also play important roles in orchestrating T_H_2-mediated immune responses. IL-8 and CCL3 are potent chemokines for neutrophils,^30^ a cell type that is associated with severe asthma.^18,19^ IL-8 is likely also secreted from other cell types in asthmatic patients, including bronchial epithelial cells. IL-21 is involved in allergic disorders, controlling the differentiation and function of T and B cells.^31^ IL-22, an IL-10 family cytokine expressed by cell types, including T_H_17, T_H_22, γδ T cells, natural killer, and group 3 innate lymphoid cells, is bifunctional, with both proinflammatory and protective effects on tissues depending on the inflammatory context. IL-22–producing cells and plasma concentrations of IL-22 are increased with the severity of atopic dermatitis and asthma.^32,33^ GM-CSF is critical for granulocyte survival and enhances their activities.^34^ Increases in GM-CSF levels are detected in patients with allergic asthma, and anti–GM-CSF antibodies administered during allergen challenge of sensitized mice inhibited airway inflammation and mucus production.^35^ Our data also confirmed that the cytokines induced by PGD_2_ and LTE_4_ from T_H_2 cells are functional, suggesting that these cytokines could make an important contribution to the downstream effects of T_H_2 cell activation. The tested cytokines (IL-8 and GM-CSF) potently promoted neutrophil activation, although neutralizing antibodies against these cytokines could not completely inhibit the neutrophil activation in response to T_H_2 supernatants, indicating other products, such as CCL3, might also contribute.

It has been recognized that numbers of neutrophils, as well as eosinophils, are increased in the sputum of patients with severe persistent asthma.^19,36,37^ The interaction between T_H_2 cells and eosinophils through type 2 cytokines has been well established. Here we reveal novel mechanisms linking T_H_2 activation and neutrophilia. Considering that upregulation of the PGD_2_ pathway and CRTH2 levels is also linked with severe and poorly controlled asthma,^27^ PGD_2_/CRTH2 might contribute to severe asthma through neutrophil recruitment and activation. A contribution of PGD_2_/CRTH2 to neutrophilic inflammation has also been demonstrated by the observation of a role of CRTH2 in contact hypersensitivity–induced skin neutrophil inflammation in the mouse.^38^

T_H_2 cells are enriched at the site of allergic inflammation, but the mechanism involved in recruitment of the cells remains obscure. Immune cells undergo a series of sequential steps during extravasation from blood to tissue, including tethering, rolling, adhesion, crawling, and transmigration.^39^ Our data suggested that PGD_2_ and LTE_4_ contribute to the T_H_2 recruitment cascade through promoting selectin-mediated rolling and integrin-dependent adhesion. Several adhesion molecules could enhance this because antibodies to CD54 and CD31 only partially reduced CAIA. The present study demonstrated the important roles of PGD_2_ and LTE_2_ in the T_H_2 cell infiltration seen during allergic inflammation.

The effect of PGD_2_ on T_H_2 cells is mediated by CRTH2 because it can be completely abolished by a selective CRTH2 antagonist but not by the inhibitor of D prostanoid receptor 1.^11^ The receptor mediating the biological activities of LTE_4_ is still uncertain, although CysLT_1_ and CysLT_2_ are both expressed by human T_H_2 cells and the effects of cysLTs, including LTE_4_, can be inhibited by the CysLT_1_ antagonist.^16,17^ The activity of LTE_4_ is unlikely to be mediated by these receptors because of their low affinity for this leukotriene compared with LTD_4_ and LTC_4_.^13,14^ The CysLT_1_-mediated calcium flux in response to cysLTs in human T_H_2 cells showed a rank order of potency as follows: LTD_4_ > LTC_4_ > LTE_4_.^16^ However, the proinflammatory efficacy of LTE_4_, used alone or in combination with PGD_2_, is much higher than that of LTD_4_ and LTC_4_.^17^ LTE_4_ can also stimulate inflammatory responses through mechanisms independent of CysLT_1_ or CysLT_2_.^28,40^ It has been proposed that both montelukast and LTE_4_ can interact with P2Y-like receptors^41,42^; however, P2Y_12_ activation is undetectable in T_H_2 cells.^16,17^ Therefore it is possible that the effect of LTE_4_ is mediated by a montelukast-sensitive receptor that is distinct from the established cysLT receptors.

In summary, this study highlights the broad proinflammatory functions of PGD_2_ and cysLTs, particularly LTE_4_, in T_H_2 cells. They combine to upregulate the expression of many proinflammatory molecules, promote cell adhesion and migration, suppress cell apoptosis, and induce neutrophil activation. These observations indicate how these 2 mast cell products can promote allergic responses and point to potential improved therapies for allergic inflammation.

**Key messages**

• The effect of PGD_2_ and LTE_4_ on activation of T_H_2 cells is much broader than previously recognized, which might contribute to the etiology of IgE/mast/T_H_2 cell–mediated allergic inflammation.
• The combination of PGD_2_ and LTE_4_ synergistically enhances proinflammatory responses in T_H_2 cells.
• The combination of PGD_2_ and LTE_4_ promotes T_H_2 cell/neutrophil crosstalk.

## Figures and Tables

**Table E1:** Primers and probes used for qPCR

Gene	Primer	Probe no.
*IL3*	5′-TTGCCTTTGCTGGACTTCA-3′ 5′-CTGTTGAATGCCTCCAGGTT-3′	60
*IL8*	5′-AGACAGCAGAGCACACAAGC-3′ 5′-ATGGTTCCTTCCGGTGGT-3′	72
*IL9*	5′-CTTCCTCATCAACAAGATGCAG-3′ 5′-AGAGACAACTGGTCACATTAGCAC-3′	59
*IL21*	5′-AGGAAACCACCTTCCACAAA-3′ 5′-GAATCACATGAAGGGCATGTT-3′	7
*IL22*	5′-CAACAGGCTAAGCACATGTCA-3′ 5′-ACTGTGTCCTTCAGCTTTTGC-3′	6
*CSF1*	5′-GCAAGAACTGCAACAACAGC-3′ 5′-ATCAGGCTTGGTCACCACAT-3′	19
*GMCSF*	5′-TCTCAGAAATGTTTGACCTCCA-3′ 5′-GCCCTTGAGCTTGGTGAG-3′	1
*GAPDH*	5′-AGCCACATCGCTCAGACAC-3′ 5′-GCCCAATACGACCAAATCC-3′	60

*CSF1*, Macrophage colony-stimulating factor 1.

**Table E2:** List of cytokines, chemokines, their receptors, and CD molecule genes regulated by PGD_2_, LTE_4_, or their combination in T_H_2 cells detected by means of microarray∗

Upregulation				Downregulation			
Gene	Sample treatment			Gene	Sample treatment		
	PGD_2_	LTE_4_	PGD_2_ + LTE_4_		PGD_2_	LTE_4_	PGD_2_ + LTE_4_
*IL1A*	+	+	++	*IL1F7*	−		
*IL3*	++	++	+++	*IL15*	−	−	−
*IL4*		+	++	*IL28B*			−
*IL5*	+++	+	+++	*IL29*			−
*IL8*	++	++	+++	*CCL4L1*	−		
*IL9*			++	*CCL28*	−		−
*IL13*	++	+	+++	*CXCL16*	−		−
*IL16*	+	+	+	*IL6R*			−
*IL22*			+	*IL7R*	−	−	−
*IL26*	+++		+++	*IL10RB*			−
*IL4I1*			+	*IL11RA*	−		−
*ILF2*			+	*IL15RA*		−	
*CSF1*	++	+	+++	*CCR2*	−	−	−
*CSF2*	+++	++	+++	*CCR3*	−	−	−
*LTA*	+		++	*CCR7*			−
*TNF*	+		++	*CXCR4*			−
*TNFSF8*	++	+	++	*CD3D*	−		−
*TNFSF10*	+	+	+	*CD40*		−	
*TNFSF11*	+	+	++	*CD47*			−
*TNFSF14*	+	+	++	*CD48*			−
*CCL1*	+	++	+++	*CD79B*	−		−
*CCL3*		++	+++	*CD80*	−		
*CCL3L1*		++	+++	*CD86*			−
*CCL3L3*		++	+++	*CD99L2*	−		
*CCL4L2*		++	+++	*CD200R1*	−		−
*CCL5*			+	*GPR44*	−		− −
*CCL15*	+	+					
*IFNGR2*	+	+	++				
*IL1RAP*		+	+				
*IL1RL1*	+++	+	+++				
*IL1RL2*	+						
*IL2RB*	+		+				
*IL2RG*	+		+				
*IL4R*		+	+				
*IL17RA*	+		+				
*IL17RB*	+						
*IL18R1*	+	+	+				
*IL18RAP*	++	++	+++				
*IL21R*	+		++				
*IL27RA*	+	+	+				
*IL28RA*			+				
*CCR4*	++		+				
*CXCR3*		+					
*CXCR5*			+				
*CXCR6*			+				
*TNFRSF8*	++		++				
*TNFRSF9*	+++	++	+++				
*CD2BP2*	+		+				
*CD5L*		+					
*CD9*	+	+	+				
*CD28*	+		+				
*CD40LG*	+		++				
*CD44*	+		+				
*CD52*	+						
*CD53*	+		+				
*CD55*			+				
*CD58*	+		+				
*CD59*			+				
*CD69*	++	++	+++				
*CD70*			+				
*CD81*			+				
*CD82*	+	+	+				
*CD83*	+		++				
*CD84*	+		+				
*CD109*	+		++				
*CD151*	+						
*CD200*			+				
*CD226*	+		+				
*CD276*	+		+				

*CSF*, Macrophage colony-stimulating factor. *++* or *− −*, Fold change of 3 or greater; *+++*, fold change of 6 or greater.

∗ Concentrations of PGD_2_ and LTE_4_ were 100 and 50 nmol/L, respectively.

**Table E3:** List of cytokine genes regulated by PGD_2_, LTE_4_, or their combination in T_H_2 cells detected by means of PCR array∗

Upregulation				Downregulation			
Gene	Sample treatment			Gene	Sample treatment		
	PGD_2_	LTE_4_	PGD_2_ + LTE_4_		PGD_2_	LTE_4_	PGD_2_ + LTE_4_
*IL3*	+	++	+++	*BMP4*	−	−	−
*IL5*	++	++	+++	*BMP8B*	−	−	−
*IL8*	++	+	+++	*GDF9*	−	−	−
*IL9*	+	+	+++	*IFNA5*	−	−	−
*IL10*	+	+	++	*IFNK*	− −	−	−
*IL13*	+	+	++	*IL15*	−	−	−
*IL21*	+	++	+++	*IL17C*	−	−	−
*IL22*	+	++	+++	*IL18*	−	−	−
*CSF1*	+	+	++	*IL24*	−	−	−
*CSF2*	++	++	+++	*NODAL*	− −	−	−
*FASLG*	++	+	++	*TGFB3*	−	−	−
*LTA*	+	+	++	*TNFSF13B*	− −	−	− −
*PDGFA*	++	+	++				
*TNF*	+	+	+				
*TNFSF8*	+	+	++				
*TNFSF10*	+	+	+				
*TNFSF11*	++	+	++				
*TNFSF14*	+	+	+				

*CSF*, Macrophage colony-stimulating factor. *++* or *− −*, Fold change of 3 or greater; *+++*, fold change of 6 or greater.

∗ Concentrations of PGD_2_ and LTE_4_ were 100 and 50 nmol/L, respectively.

**Table I:** List of genes encoding adhesion molecules upregulated by PGD_2_, LTE_4_, or their combination in T_H_2 cells detected by using a microarray∗

Gene	Protein	Sample treatment		
		PGD_2_	LTE_4_	PGD_2_ + LTE_4_
*FBLN7*	Fibulin 7	++	+	++
*IGSF3*	Immunoglobulin superfamily, member 3	+		++
*CTNNAL1*	Catenin alpha-like 1	++		++
*CTNNA1*	Catenin alpha-1	+	+	++
*NINJ1*	Ninjurin-1	+		++
*CEACAM1*	CD66a	+		+
*PECAM1*	Platelet endothelial cell adhesion molecule	+	+	+
*CD226*	CD226 molecule	+	+	+
*CD9*	CD9 molecule	+	+	+
*DCHS1*	Dachsous 1			+
*ICAM1*	CD54	+		+
*PCDHA4*	Protocadherin alpha-4			+
*LAMA5*	Laminin, alpha 5	+		+
*SELE*	E-selectin			+
*ITGAV*	Integrin alpha-V		+	+
*ITGAX*	CD11c	+		+
*CD44*	CD44 molecule	+		+
*ITGA2*	CD49b			+
*ITGB1BP1*	Integrin beta-1–binding protein 1	+		+
*CTNND1*	Catenin delta-1		+	+
*ICAM2*	CD102			+
*CIB2*	Calcium and integrin binding family member 2			+
*PCDHA1*	Protocadherin alpha-1			+
*CD151*	CD151 molecule (Raph blood group)	+		
*ITGAE*	Integrin, alpha E	+		
*CDH1*	Cadherin-1	+		
*ITGA11*	Integrin alpha-11		+	

*++*, Fold change greater than 3.

∗ The concentrations of PGD_2_ and LTE_4_ were 100 and 50 nmol/L, respectively.

## Methods

### Reagents

PGD_2_ and LTE_4_ were purchased from Enzo Life Science (Farmingdale, NY). TM30089 was supplied by ChemieTek (Indianapolis, Ind). The human CD4^+^ T Cell Isolation Kit II, anti-human CRTH2 MicroBead Kits, T-cell activation/expansion kits, and anti-human CRTH2 antibody were from Miltenyi Biotec (Bergisch Gladbach, Germany). X-VIVO 15 medium was purchased from Lonza. AIM V medium was purchased from Invitrogen (Carlsbad, Calif). HBSS was from Gibco (Carlsbad, Calif). Ficoll-Paque Plus was supplied by GE Healthcare (Pittsburgh, Pa). Lymphoprep was purchased from Axis-Shield UK (Dundee, United Kingdom). The RNeasy Mini kit and Omniscript Reverse Transcription kit were supplied from Qiagen. Real-time qPCR Master Mix and probes were from Roche. Primers were synthesized by Eurofins MWG Operon (Ebersberg, Germany). Human rIL-8, anti–IL-8, and anti-CD54 (ICAM-1) antibodies were purchased from R&D Systems. Anti-CD31 (PECAM-1) antibody was from Thermo Fisher Scientific (Waltham, Mass). Anti-human GM-CSF, CD3, CD4, CCR6, CCR7, CD45RO, IL-4, IL-5, IL-8, IL-13, and IFN-γ antibodies and Annexin V–allophycocyanin were obtained from BioLegend (San Diego, Calif). Anti-human CD16, IL-22, and GATA3 antibodies and the viability dye eFluor 780 were from eBioscience (San Diego, Calif). Anti-human RORγt antibody was from BD Biosciences. Human rIL-2, human rIL-4, and human rGM-CSF were from PeproTech (Rocky Hill, NJ). Other chemicals were from Sigma-Aldrich (St Louis, Mo).

### T_H_2 cell preparation

Human CD4^+^CRTH2^+^ T_H_2 cells were prepared, as described in our previous reports.^E1^ Briefly, PBMCs were isolated from buffy coats by using Ficoll-Paque Plus density gradient centrifugation, followed by CD4^+^ cell purification with the MACS CD4^+^ T Cell Isolation Kit II. After a 7-day culture in AIM V medium containing 10% human serum, 50 U/mL rhIL-2, and 100 ng/mL rhIL-4, CRTH2^+^ cells were isolated from the CD4^+^ culture by means of positive selection with the anti-human CRTH2 MicroBead Kit. The harvested CD4^+^/CRTH2^+^ cells were treated as T_H_2 cells and further amplified in X-VIVO 15 medium containing 10% human serum and 50 U/mL rhIL-2 before use. For *ex vivo* T_H_2 cells, CRTH2^+^ cell isolation was directly conducted after CD4^+^ cell purification without culture.

### Flow cytometric analysis for T_H_2 cell phenotype

For surface marker staining, T_H_2 cells or PBMCs from fresh blood were fluorescently labeled with antibodies to CD3, CD4, CRTH2, CCR6, CCR7, CD45RA, and CD45RO in PBS containing 0.2% BSA and 2 mmol/L EDTA and then fixed with 2% formaldehyde. Transcription factor staining was conducted in permeabilization buffer with 0.05% saponin-containing antibodies to GATA3 and RORγt. For analysis of intracellular cytokines, T_H_2 cells were stimulated with PMA (5 ng/mL) and ionomycin (500 ng/mL) for 4 to 5 hours. Brefeldin A (5 μg/mL) was added at 30 minutes after starting stimulation. Then the cells were stained in a permeabilization buffer containing antibodies to IFN-γ, IL-4, IL-5, IL-8, IL-13, and IL-22. Nonstimulated cells were used as a negative control, and dead cells were excluded by using the viability dye eFluor 780. After staining, the cells were acquired with an LSR II Flow Cytometer (BD Biosciences).

### Western blotting

T_H_2 cells were treated for 15 minutes. Cell lysis and Western blotting were performed, as described previously.^E2^ Briefly, the cells were solubilized in lysis buffer (20 mmol/L Tris-HCl [pH 7.4], 250 mmol/L sucrose, 1 mmol/L EDTA, 1 mmol/L ethyleneglycol-bis-[β-aminoethylether]-N,N,N′,N′-tetraacetic acid, 1 mmol/L sodium orthovanadate, 10 mmol/L sodium glycerophosphate, 50 mmol/L sodium fluoride, 5 mmol/L sodium pyrophosphate, 0.1% 2-mercaptoethanol, protease inhibitor mixture, and 1% Triton X-100). The samples were fractionated by using SDS-PAGE and then electrophoretically transferred to a nitrocellulose membrane and probed with antibodies, as indicated in the Results section. The intensity of immunopositive bands was quantified by using ImageJ software (National Institutes of Health, Bethesda, Md).

### Quantification of cell aggregates

Briefly, cell clumps were identified by using the Otsu method of thresholding. The intensity and size of identified objects were measured. Results are reported as integrated intensity.
